# Dataset of accumulated internal gas pressure and temperature during lithium-ion battery operation and ageing

**DOI:** 10.1016/j.dib.2025.111420

**Published:** 2025-02-26

**Authors:** Begum Gulsoy, Timothy Vincent, Calum Briggs, Ashima Kalathingal, Mona Faraji Niri, James Marco

**Affiliations:** WMG, University of Warwick, Coventry, CV4 7AL, United Kingdom

**Keywords:** Gas pressure, In-situ monitoring, Ageing, Internal cell-sensing, Advanced characterisation

## Abstract

The experimental data presented are relates to the research article entitled “in-situ measurement of internal gas pressure within cylindrical lithium-ion cells” [1]. In brief, internal gas pressure that provides deeper insights into the reversible and irreversible gas generation inside a lithium-on cell was directly measured using a novel bespoke embedded sensor system during cell operation and long-term ageing. Battery performance assessment data was obtained from reference performance tests (RPTs) conducted after each instrumentation stages (defined in [1] as: pristine, modified and instrumented conditions) and at 20-cycle ageing intervals, while ageing data was collected over a total of 100 cycles. Key characterisation parameters, such as cell voltage, discharge capacity at a current of 1C discharge, direct current internal resistance (DCIR) at different states of charge (100 %, 80 % and 50 % SOC), cell surface temperature and internal gas temperature were recorded using instrumented commercial cylindrical cells (LG-Chem INR21700-M50) with embedded pressure sensors. This data provides insights into gas generation within cylindrical cells and demonstrates the inherent coupling between state of charge (SOC), degradation and temperature and pressure variation. The published data provides valuable resources for enhanced battery diagnostics and development of data-driven models to estimate state of charge and state of health (SOH) for advanced battery safety monitoring and BMS control systems.

Specifications Table

**Table:** 

Subject	Electrochemistry
Specific subject area	Characterization of lithium-ion batteries by internal gas pressure and temperature
Type of data	Processed
Data collection	Cell characterisation parameters such as cell voltage, discharge capacity and direct current internal resistance (DCIR) were recorded using Bio-Logic VSP-300 Potentiostat. Cell energy capacity was recorded at a current of 1C discharge. DCIR values were derived from 2C pulse discharges conducted at 100 %, 80 % and 50 % SOC. Furthermore, cell surface temperature and internal gas pressure were logged using TC-08 PicoLog and Omega Digital Transducer Application, respectively. Data sample rates were set to 10 Hz. All data were collected after 2-hour period of temperature equilibrium inside Binder climatic chamber set to 25 °С. All data were processed using MATLAB 2024a analytical software.
Data source location	Institution: Warwick Manufacturing Group (WMG), Energy Innovation Centre, University of Warwick City: Coventry Country: United Kingdom GPS coordinates for collected samples/data: 52.38363378953185, −1.5615186436655097
Data accessibility	Repository name: Mendeley Data Data identification number: 10.17632/pn5ct66rn5.1 Direct URL to data: https://data.mendeley.com/datasets/pn5ct66rn5/1
Related research article	Gulsoy, B., Vincent, T.A., Briggs, C., Sansom, J.E. and Marco, J., 2023. In-situ measurement of internal gas pressure within cylindrical lithium-ion cells. Journal of Power Sources, 570, p.233064. https://www.sciencedirect.com/science/article/pii/S0378775323004391

## 1 Value of the Data

• The presented data improves our fundamental understanding of gas accumulation inside the cylindrical cells during operation and ageing. The data of gas pressure within cylindrical cells (LG-Chem INR21700-M50) are not publicly available and therefore the presented data fills a currently existing gap in the publicly available dataset.
• The data provides insights into the effects of state of charge (SOC), degradation and temperature changes on gas pressure accumulation. This data can be further utilised to investigate how the accumulation of gas pressure varies as a function battery SOH and if there is any corelation with specific degradation modes, often defined as a loss of active material (LAM) or Loss of Lithium Inventory (LLI). The data also provides insights into the potential mechanical deformation of the electrodes within the cell during charge-discharge.
• This data can support future innovations in battery diagnostics, and to support the parameterisation and validation of electrochemical models for advanced battery safety and BMS control systems.

## 2 Background

The performed experiments aim to increase our level of fundamental understanding of gas generation during lithium-ion battery operation by investigating the effects of state of charge (SOC), degradation and temperature changes on gas pressure accumulation. Gas generation is influenced by factors such as the composition of electrolyte, the nature of the electrodes (e.g. the anode), cycling and operating conditions, e.g., cut-off voltage and temperature. Gas accumulates inside the batteries, causing increasing resistance, further heat generation and performance loss. More importantly it is known to increases the mechanical stress in the electrode materials, leading to accelerated degradation and potential safety risks. Therefore, the gas pressure dataset can be used for various applications such as enhanced battery diagnostics, formation of data-driven models to estimate state of charge (SOC) and state of health (SOH), and advanced battery design. Additionally, this dataset enhances the reproducibility of the findings in [1], developing scientific collaboration and innovation. This initiative aims to advance knowledge and promote cooperation in the pursuit of sustainable and safer energy solutions.

## 3 Data Description

The experimental dataset comprises three instrumented LG-Chem INR21700-M50 cells, subjected to an aging protocol of 100 cycles, with a reference performance test (RPT) conducted at intervals of 20 cycles. The technical specifications of the cells are summarised in Table 1.

**Table 1:** Technical specification for LG-Chem INR21700-M50.

Item	Specification/Value/Material	Unit
Format	21700	−
Rated capacity	5	Ah
Mass	68.0 ± 1.0	g
Nominal voltage	3.63	V
Charge cut-off voltage	4.2	V
Discharge cut-off voltage	2.5	V
Operating temperature	Charge: 0 ∼ +45	°С
	Discharge: -20 ∼ +60	°С
Cathode	LiNiMnCoO_2_	−
Anode	Graphite - SiO_x_	−
Separator	Ceramic-coated	−

**Fig. 1:**
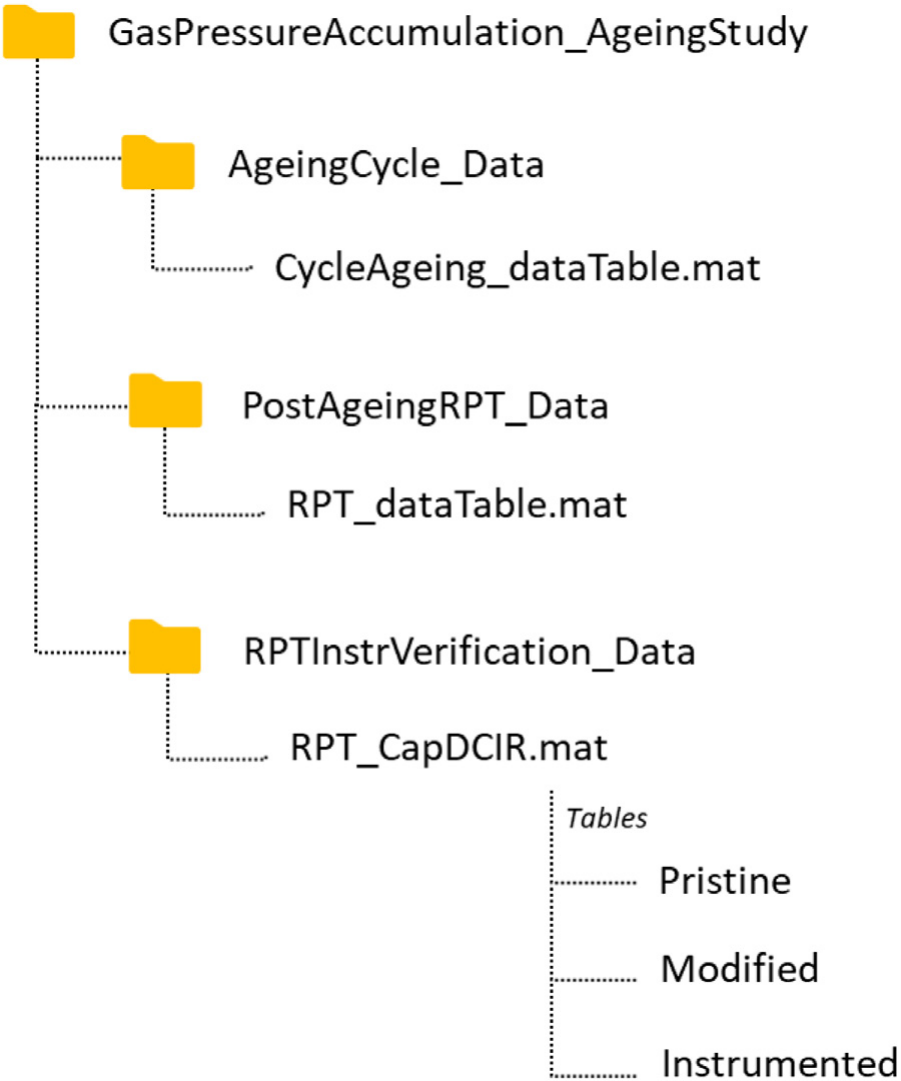
The overall file structure of the dataset.

The overall file structure of the dataset is shown in Fig. 1. Within the parent folder *GasPressureAccumulation_AgeingStudy*, there are three subfolder named *AgeingCycle_Data, PostAgeingRPT_Data* and *RPTInstrVerication_Data*. These subfolders contain: (i) the combined dataset collected over a total of 100 cycles; (ii) five RPTs conducted at 20-cycle intervals, and (iii) the RPTs conducted after each instrumentation stages for process verification, respectively. The first two subfolders each contain a single data table, while the third subfolder contains three data tables corresponding to datasets for the pristine, modified, and instrumented conditions.

The parameters of *CycleAgeing_dataTable* and *RPT_dataTable* datasets represent raw data collected from the cyclers and the sensors through data loggers. These tables contain parameters specifying the cells and sensors type, such as *CellID, Condition, SensorID, CellManufacturer* and *CellType*. Moreover, the data tables include various cell parameters from the ageing experiments. These parameters as follows: *Mode*, indicating experimental stages in the test protocol; *TestTime*, representing experimental duration (in seconds in *CycleAgeing_dataTable* and in yyyy-mm-dd hh:mm:ss.ss format in *RPT_dataTable*); *Voltage*, indicating the cell voltage; *Current*, illustrating the applied current to the cells; *Qcharge* and *Q discharge*, representing capacity changes during charging and discharging stages, respectively; *Capacity*, showing the total cell capacity; *Resistance*, illustrating cell resistance; *Pressure*, representing the gas pressure changes inside the cells relative to atmospheric pressure; *SurfaceTemperature*, illustrating the cell surface temperature.

**Fig. 2:**
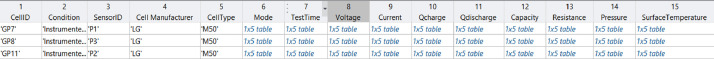
The data table structure and recorded parameters for CycleAgeing_dataTable and RPT_dataTable.

The table structure and the parameters are shown in Fig. 2. From Column *Mode* to *SurfaceTemperature*, each cell contains a “1 × 5 table” representing the 20-cycle intervals. These are as follows:

• C1 (0 - 20 cycles)
• C2 (21 - 40 cycles)
• C3 (41 - 60 cycles)
• C4 (61- 80 cycles)
• C5 (81 - 100 cycles)

*RPT_CapDCIR* dataset contains measured and calculated values derived from the equations explained in Calculations. The parameters of *RPT_CapDCIR* are as follows: as *CellID*, identifying the cell ID; *Cap,* showing changes in cell capacity after a specified of cycles; *R100, R80, R50* and *R20,* representing the direct current internal resistance (DCIR) at 100 %, 80 %, 50 % and 20 % state of charge (SOC), respectively.

## 4 Experimental Design, Materials and Methods

All cycling and characterisation experiments are performed within a climate chamber (Binder MKF56) using Bio-Logic VSP-300 Potentiostat. Defined test protocols are designed with EC-Lab software. Table 2 shows the ageing protocol, including of a total of 100 cycles, and Table 3 illustrates the reference performance test (RPT) protocol conducted after each 20-cycle. Cell surface temperature was monitored by the K-type thermocouples (363-0250, RS Pro) attached to the centre location of the cell. Temperature data was logged using a PicoLog (TC-08). Internal gas pressure was monitored by a digital Gauge pressure transducer (MM-G-100-USBH-B-4-MD-0-T8-A3-CE, Omega), and the data logged by the Omega Digital Transducer Application.

**Table 2:** Ageing protocol.

Step	Mode	Action	Limits	Record	Description
1	Rest		Time: 1 h	1 s	Temperature control
2	Discharge	C/3 = 1.67 A	V < 2.5 V	1 s, 10 mV	C/3 Discharge to 0 % SOC
3	Rest		Time: 1 s	1 s	
4	Charge	CC C/3 = 1.67 A	V < 4.2 V	1 s, 10 mV	C/3 Charge to 100 % SOC
		CV 4.2 V	I < C/20 = 0.25 A	1 s, 10 mV	
5	Discharge	CC 1 C = 5 A	Time: 30 mins	1 s, 10 mV	1C Discharge to 0 % SOC
6	Loop	Go to Step 3	20 times		Repeat cycle
7	Reference Performance Test	(See Table 3)

**Table 3:** Reference performance test (RPT) protocol.

Step	Mode	Action	Limits	Record	Description
1	Rest		Time: 1 h	1 s	Temperature control
2	Discharge	C/3 = 1.67 A	V < 2.5 V	1 s, 10 mV	Discharge to 0 % SOC
3	Rest		Time: 30 mins	1 s	
4	Charge	CC C/3 = 1.67 A	V < 4.2 V	1 s, 10 mV	Charge to 100 % SOC
		CV 4.2 V	I < C/20 = 0.25 A	1 s, 10 mV	
5	Rest		Time: 30 mins	1 s	
6	Discharge	1 C = 5 A	V < 2.5 V	1 s, 10 mV	Initial cell capacity at 1C
7	Rest		Time: 1 s	0.1 s	
8	Rest		Time: 1 h	1 s	
9	Discharge	C/10 = 0.5 A	V < 2.5 V	1 s, 10 mV	Residual capacity at C/10
10	Rest		Time: 1 s	0.1 s	
11	Rest		Time:1 h	1 s	
12	Charge	CC C/3 = 1.67 A	V < 4.2V	1 s, 10 mV	Charge to 100 % SOC
		CV 4.2 V	I < C/20 = 0.25 A	1 s, 10 mV	
13	Rest		Time: 1 h	1 s	
14	Discharge	2 C = 10 A	Time: 30 s	0.1 s	2C pulse discharge at 100 % SOC
15	Rest		Time: 1 min	0.1 s	
16	Rest		Time: 19 mins	1 s	
17	Charge	C/3 = 1.67 A	Time: 3 mins	1 s, 10 mV	Compensation of capacity loss
18	Rest		Time: 20 mins	1 s	
19	Discharge	C/2 = 2.5 A	Time: 30 s	0.1 s	C/2 pulse discharge at 100 % SOC
20	Rest		Time: 1 min	0.1 s	
21	Rest		Time: 19 mins	1 s	
22	Charge	C/3 = 1.67 A	Time: 45 s	0.1 s, 10 mV	Compensation of capacity loss
23	Rest		Time: 20 mins	1 s	
24	Discharge	C/3 = 1.67 A	Time: 36 mins	1 s	100% to 80% SoC
25	Rest		30 mins	1 s	
26	Discharge	2 C = 10 A	Time: 30 s	0.1 s	2C pulse discharge at 80 % SOC
27	Rest		1 min	0.1 s	
28	Rest		19 mins	1 s	
29	Charge	C/3 = 1.67 A	Time: 3 mins	1 s, 10 mV	Compensation of capacity loss
30	Rest		Time: 20 mins	1 s	
31	Discharge	C/2 = 2.5 A	Time: 30 s	0.1 s	C/2 pulse discharge at 80 % SOC
32	Rest		Time: 1 min	0.1 s	
33	Rest		Time: 19 mins	1 s	
34	Charge	C/3 = 1.67 A	Time: 45 s	1 s, 10 mV	Compensation of capacity loss
35	Rest		Time: 20 mins	1 s	
36	Discharge	C/3 = 1.67 A	Time: 54 mins	1 s	80% to 50% SoC
37	Rest		Time: 30 mins	1 s	
38	Discharge	2 C = 10 A	Time: 30 s	0.1 s	2C pulse discharge at 50 % SOC
39	Rest		Time: 1 min	0.1 s	
40	Rest		Time: 19 mins	1 s	
41	Charge	C/3 = 1.67 A	Time: 3 mins	1 s, 10 mV	Compensation of capacity loss
42	Rest		Time: 20 mins	1 s	
43	Discharge	C/2 = 2.5 A	Time: 30 s	0.1 s	C/2 pulse discharge at 50 % SOC
44	Rest		Time: 1 min	0.1 s	
45	Rest		Time: 19 mins	1 s	
46	Charge	C/3 = 1.67 A	Time: 45 s	1 s, 10 mV	Compensation of capacity loss
47	Rest		Time: 20 mins	1 s	
48	Discharge	C/3 = 1.67 A	Time: 54 mins	1 s	50% to 20% SoC
49	Rest		Time: 30 mins	1 s	
50	Discharge	2 C = 10 A	Time: 30 s	0.1 s	2C pulse discharge at 20 % SOC
51	Rest		Time: 1 min	0.1 s	
52	Rest		Time:19 mins	1 s	
53	Charge	C/3 = 1.67 A	Time: 3 mins	1 s, 10 mV	Compensation of capacity loss
54	Rest		Time: 20 mins	1 s	
55	Discharge	C/2 = 2.5 A	Time: 30 s	0.1 s	C/2 pulse discharge at 20 % SOC
56	Rest		Time: 1 min	0.1 s	
57	Rest		Time: 19 mins	1 s	
58	Charge	C/3 = 1.67 A	Time: 45 s	1 s, 10 mV	Compensation of capacity loss
59	Rest		Time: 1 h	1 s	
60	Discharge	C/3 = 1.67 A	Time: 31 mins	1 s, 10 mV	20% to 3% SoC
61	Rest		Time: 1 h	1 s	

## 5 Calculations

**Fig. 3:**
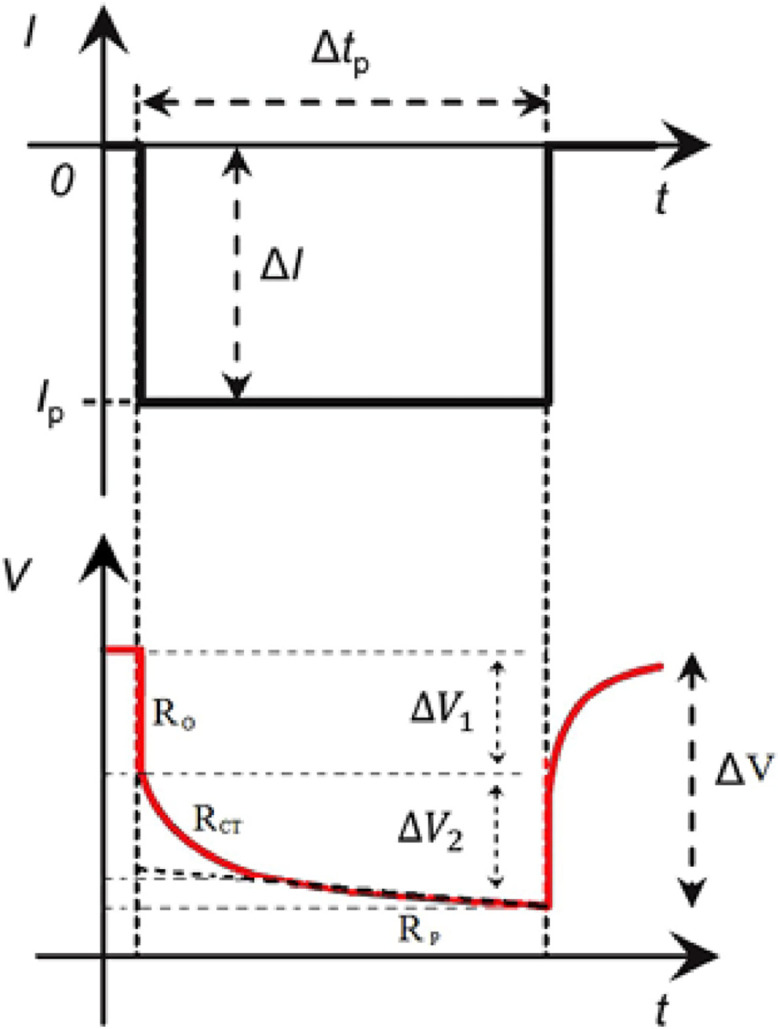
Cell voltage response to a pulse current [2].

Direct current internal resistance (DCIR) of a battery has been calculated using Eq. (1). In the equation, $\Delta V_{1}$ is the instantaneous voltage changes due to the pure ohmic resistance, and ΔI is the pulse current perturbation, as shown in Fig. 3. DCIR has been calculated for the 2C pulse discharges at 100%, 80%, 50% and 20% SOC.

(1) $$\text{DCIR}\;\left[\Omega \right]=\;\frac{\Delta V_{1}\left(V\right)}{\Delta I_{\;}\left(A\right)}$$

## Limitations

Not applicable.

## Ethics Statement

The proposed data does not involve any human subjects, animal experiments, or data collected from social media platforms. The authors confirm that this work meets the ethical requirements of the journal*.*

## Credit Author Statement

B. Gulsoy: Conceptualization, Methodology, Formal analysis, Investigation, Writing - Original Draft. T.A. Vincent: Writing - Review & Editing, Investigation. C. Briggs: Methodology, Investigation. A. Kalathingal: Data Processing. M.F. Niri: Data Processing, Writing - Review & Editing. J. Marco: Funding acquisition, Supervision, Resources, Writing - Review & Editing.

## Data Availability

Mendeley DataDataset of accumulated internal gas pressure and temperature during lithium-ion battery operation and ageing (Original data)
